# Non-occlusive Mesenteric Ischemia Following Severe Traumatic Brain Injury: A Case Report

**DOI:** 10.7759/cureus.79146

**Published:** 2025-02-17

**Authors:** Kazuki Fujii, Kiyoshi Takemoto, Shiho Yoshida, Keisuke Takano, Kazuaki Atagi

**Affiliations:** 1 Emergency and Critical Care Medicine, Nara Prefecture General Medical Center, Nara, JPN

**Keywords:** gut-brain-axis, non-occlusive mesenteric ischemia, onuf's nucleus, risk factor, traumatic brain injury

## Abstract

Although various risks associated with non-occlusive mesenteric ischemia (NOMI) have been reported, to the best of our knowledge, no report has suggested an association between severe traumatic brain injury (sTBI) and NOMI. We experienced a case of NOMI in a young woman with no risk except sTBI. A passerby discovered an unresponsive woman lying on the street and summoned emergency medical services. The woman was subsequently transported to our emergency department by ambulance. The patient was diagnosed with sTBI and underwent emergency craniotomy on the same day. After surgery, she was placed under general management in the intensive care unit (ICU). She was discharged from the ICU on postoperative day 9. On postoperative 14, she experienced sudden cardiac arrest. Based on clinical findings, cardiac arrest in this case was attributed to hypoxia due to aspiration caused by abdominal distension and vomiting following the onset of NOMI. This case report suggests that sTBI may be a risk factor for the development of NOMI.

## Introduction

Non-occlusive mesenteric ischemia (NOMI) is an acute mesenteric circulatory disorder that occurs without organic vascular occlusion and is associated with extremely high mortality [1]. The known risk factors for NOMI include advanced age, renal disease, diabetes mellitus, decreased cardiac output, vasopressor administration, intra-aortic balloon pump, and increased inflammatory markers [2-4]. The majority of patients with NOMI are elderly. There are few reports of NOMI in younger patients [5,6], but these cases were reported either after cardiac arrest or as a result of hemorrhage or dehydration. However, a 46-year-old woman with no risk except for severe traumatic brain injury (sTBI) developed NOMI. To the best of our knowledge, there have been reports of NOMI in patients with central nervous system disorders [7], but none specifically associated with sTBI. We report a case in which TBI was suspected to be involved in the development of NOMI.

## Case presentation

A passerby discovered an unresponsive 46-year-old woman lying on the street and summoned emergency medical service. The woman was subsequently transported to our emergency department by ambulance. Her past medical history was unremarkable. She had no allergies to any medications or food. Her vital signs were as follows: Glasgow Coma Scale score of 10 (E4V1M5), blood pressure of 150/122 mmHg, heart rate of 66 beats per minute (sinus rhythm), respiratory rate of 17 breaths per minute, blood oxygen saturation of 100% on ambient air, and body temperature of 35.7°C. Physical examination revealed a subcutaneous hematoma and contusion on the right temporal area and raccoon eye in the right eye. Laboratory data on visiting the hospital was unremarkable (hemoglobin 14.1 g/dL, aspartate aminotransferase 41 U/L, alanine aminotransferase 20 U/L, lactate dehydrogenase 324U/L, creatinine kinase 394U/L, blood urea nitrogen 16.3mg/dL, and creatinine 0.62 mg/dL).

**Figure 1:**
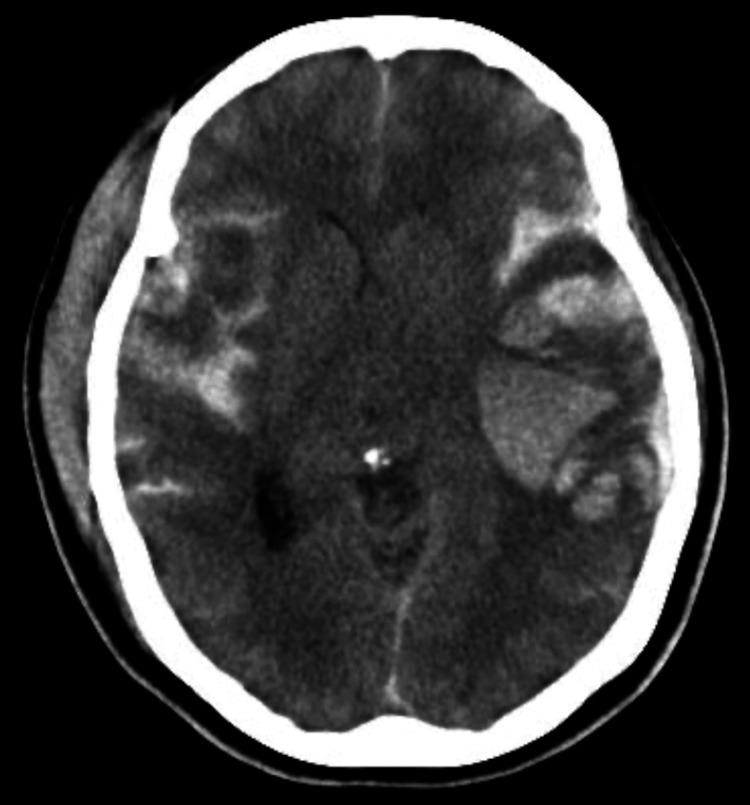
Head computed tomography (CT) scan two hours after arrival Two hours later, the CT scan showed an enlarged contusional hematoma in the left cerebrum and a midline shift.

A head computed tomography (CT) scan revealed an extensive subcutaneous hematoma centered on the right frontal area, a left acute subdural hematoma, extensive cerebral contusion, and a fracture of the right temporal bone. There were no significant findings in the CT scan of the trunk. In addition, there was no evidence of mesenteric injury on this CT. At this point, we determined that emergency craniotomy was not indicated and decided to follow up with CT two hours later. Two hours later, the CT scan showed an enlarged contusional hematoma in the left cerebrum and midline shift (Figure 1), so we decided to perform cranial decompression surgery to save the patient’s life. After surgery, the patient was admitted to the ICU for management to prevent secondary brain injury. In addition, we managed the patient while paying attention to her enteral nutrition, which is a risk factor for NOMI. Her general condition was stable. A small amount of enteral nutrition was initiated on postoperative day 1, and it was gradually increased. A tracheostomy was performed on postoperative day 6. Intestinal blood flow was carefully monitored, and patient management aimed to prevent excessive negative balance and rapid fluctuations in blood pressure. She was discharged from the intensive care unit (ICU) on postoperative day 9. Upon ICU discharge, her general condition was unremarkable except for her level of consciousness (E2VTM5). She defecated normally and enteral feeding was titrated steadily. Her bowel movements were normal.

**Figure 2:**
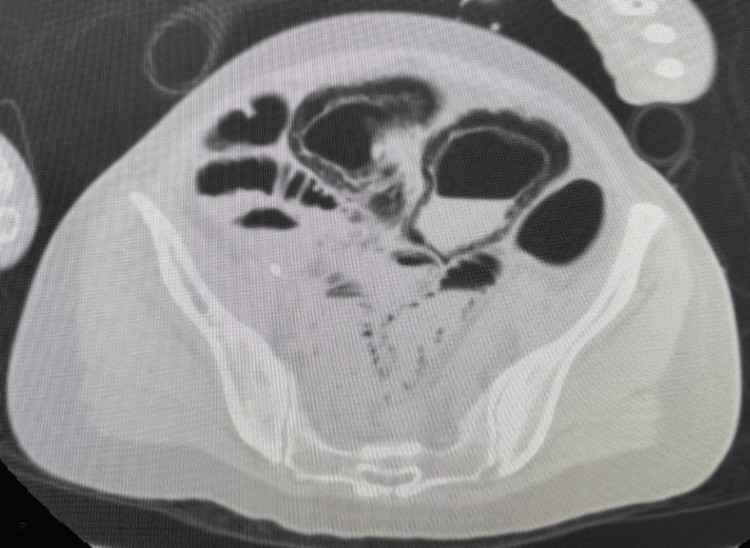
Abdominal computed tomography (CT) scan The CT scan showed no significant findings in the head; however, in the trunk, portal gas and intestinal dilatation from the jejunum to the sigmoid colon and intramural emphysema were observed.

On postoperative day 14, she suddenly vomited and developed respiratory failure due to aspiration, leading to cardiac arrest. The first responder promptly initiated cardiopulmonary resuscitation, and a return of spontaneous circulation was confirmed 19 minutes after her collapse. We performed a whole-body CT scan to search for the cause of cardiac arrest. The CT scan showed no significant findings in the head; however, in the trunk, portal gas and intestinal dilatation from the jejunum to the sigmoid colon and intramural emphysema were observed (Figure 2). No free air was evident in the CT scan. Based on this finding, we considered the possibility of intestinal ischemia to be high. Although the lack of a contrast-enhanced CT scan and pathological confirmation precluded a definitive diagnosis of NOMI, we considered that she developed NOMI based on her risk factors. Laboratory data at the time of cardiac arrest were as follows: hemoglobin, 9.0 g/dL; aspartate aminotransferase, 1,759 U/L; alanine aminotransferase, 1,429 U/L; lactate dehydrogenase, 3,770 U/L; creatine kinase, 212 U/L; blood urea nitrogen, 41.7 mg/dL; and creatinine, 1.61 mg/dL. She was not hemodynamically stable and died the same day.

## Discussion

NOMI is considered to be caused by various factors leading to mesenteric vasoconstriction, which in turn leads to ischemia and necrosis of the intestinal tract despite the absence of organic obstruction of the mesenteric vessels [3]. Various factors have been reported to cause mesenteric vasoconstriction [3,4], but no case report has suggested any relationship between mesenteric vasoconstriction and sTBI. In addition, most patients who develop NOMI are elderly. Therefore, the involvement of various factors in the development of NOMI cannot be denied, and it is quite difficult to examine the factors. Moreover, as it is not easy for us to diagnose NOMI at an early stage, we believe that the best prevention is to be aware of the risk factors for NOMI in our practice. That is why we thought it was important to look at the risk factors for NOMI. There have been several reports of NOMI in young patients [5,6], but these have occurred either after cardiac arrest or following hemorrhage or severe dehydration.

Based on various clinical findings, we speculate that the cause of cardiac arrest in this case was hypoxia due to aspiration caused by abdominal distention and vomiting after the onset of NOMI. The patient had no prior history of trauma, abdominal and cerebral disabilities, or any vascular risk factors including diabetes, dyslipidemia, arterial fibrillation, and hypertension. Her hemodynamics were stable throughout. We also adjusted for early enteral feedings, noting that this could be a risk for NOMI, particularly after its initiation. Careful observation is essential for the early detection of NOMI by monitoring the amount of nutrition remaining in the stomach and the peristalsis of the bowel, assessing ascites through abdominal echography, and confirming that there are no findings suggestive of intestinal ischemia on blood tests. Considering that women in their 40s who were not at risk of developing NOMI except for sTBI developed NOMI, we hypothesized that sTBI was involved in the development of NOMI.

Here, we discuss the relationship between the central nervous system network and intestinal/mesenteric vessels. Studies on defecation and urination have clarified the relationship between the cerebral cortex and Onuf's nucleus. A neural network between the cerebral cortex and Onuf's nucleus has been reported [8]. The Onuf’s nucleus in the sacral medulla innervates a network of sacral parasympathetic nerves. Sacral parasympathetic nerves innervate the intestinal and mesenteric vessels. Animal studies suggest that ischemia of the Onuf’s nucleus, induced by subarachnoid hemorrhage (SAH), can lead to mesenteric vasoconstriction mediated by the parasympathetic nervous system [9]. Furthermore, when a significant hemispheric lesion causes disrupted blood flow at the base of the middle cerebral artery for an hour, it can result in several effects, including the loss of cholinergic innervation in the ileum, heightened gut permeability, intestinal paralysis, and increased sympathetic activity [10]. In this case, we considered two possible mechanisms for the development of NOMI. First, we hypothesized that the sTBI may have affected the sacral parasympathetic network, which may have affected the mesenteric vasoconstriction. The second hypothesis is that the gut-brain axis may have caused NOMI. We suggest that the additional stress on the head caused by brain injury may put stress on the nervous system and hormones, causing the mesentery to spasm. There are some limitations to this case report. This means that in this case, we were unable to perform a pathological examination.

## Conclusions

Since various factors are involved in the development of NOMI, it is tough to determine its cause. However, in the present case, no risk was observed other than sTBI, suggesting that sTBI was involved in the development of NOMI.

## References

[1] Suzuki S, Kondo H, Furukawa A, Kawai K, Yamamoto M, Hirata K (2015). The treatment and diagnosis of non-occlusive mesenteric ischemia (NOMI). J Abdom Emerg Med.

[2] Lim JY, Kim JB, Jung SH, Choo SJ, Chung CH, Lee JW (2017). Risk factor analysis for nonocclusive mesenteric ischemia following cardiac surgery: a case-control study. Medicine (Baltimore).

[3] Klotz S, Vestring T, Rötker J, Schmidt C, Scheld HH, Schmid C (2001). Diagnosis and treatment of nonocclusive mesenteric ischemia after open heart surgery. Ann Thorac Surg.

[4] Stöckmann H, Roblick UJ, Kluge N (2000). Diagnosis and therapy of non-occlusive mesenteric ischemia (NOMI) [Article in German]. Zentralbl Chir.

[5] Sato T, Hasegawa S, Tezuka K, Mizutani M, Isobe H, Hachiya O, Kimura W (2011). A case of non-occlusive shock-related mesenteric ischemia due to uterine rupture. J Abdom Emerg Med.

[6] DiMeglio LA, Chaet MS, Quigley CA, Grosfeld JL (2003). Massive ischemic intestinal necrosis at the onset of diabetes mellitus with ketoacidosis in a three-year-old girl. J Pediatr Surg.

[7] Fujii K, Seki T, Nakata Y, Atagi K, Matsuyama T (2022). Non-occlusive mesenteric ischemia during acute stroke management: three case reports. Surg Case Rep.

[8] Callaghan B, Furness JB, Pustovit RV (2018). Neural pathways for colorectal control, relevance to spinal cord injury and treatment: a narrative review. Spinal Cord.

[9] Karadeniz E, Caglar O, Firinci B (2019). Predeterminative role of Onuf's nucleus ischemia on mesenteric artery vasospasm in spinal subarachnoid hemorrhage: a preliminary experimental study. Asian J Surg.

[10] Gulrandhe P, Acharya S, Shukla S, Patel M (2023). Neuropsychiatric and neurological diseases in relation to the microbiota-gut-brain axis: from research to clinical care. Cureus.

